# Assessment of a Geriatric Evaluation and Management in the Home (GEMITH) Service at a Quaternary Hospital: A Retrospective Observational Study

**DOI:** 10.1177/08971900241262376

**Published:** 2024-06-13

**Authors:** Keshia R. De Guzman, Duncan Long, Alexander Theodos, Alexandra Karlovic, Nazanin Falconer

**Affiliations:** 1 Pharmacy Department, 1966 Princess Alexandra Hospital, Brisbane, QLD, Australia; 2 School of Pharmacy, 104824 The University of Queensland, Brisbane, QLD, Australia

**Keywords:** geriatrics, medication safety, transitions of care

## Abstract

**Background:** The increasing aging population in Australia has created a higher demand for specialist geriatric services within hospitals. A Geriatric Evaluation and Management in the Home (GEMITH) service was implemented at a quaternary Queensland hospital. The GEMITH service was unique as it incorporated a specialist pharmacist into the multidisciplinary team. **Objective:** To determine the medication safety and quality impact of the GEMITH service by evaluating the type and clinical significance of specialist pharmacist interventions. **Methods:** This was retrospective observational study of clinical interventions made by the GEMITH pharmacist for patients admitted to the service between October 2020 to April 2021. All pharmacist interventions were rated for their clinical significance using the Society of Hospital Pharmacists of Australia (SHPA) risk classification system. The ratings were undertaken by a panel of three pharmacists that independently assessed the interventions, coming together for final discussion. A narrative analysis of the interventions were derived through group consensus. **Results:** There was a total of 119 admissions to the GEMITH service, with 132 clinical interventions made by the specialist geriatric pharmacist. The majority (47%) of interventions were considered as low risk interventions, although high- (21%) and extreme-risk (2%) interventions still occurred. The most common type of intervention (32%) involved medication reconciliation. Other intervention types included monitoring recommendations, dosing interventions, and deprescribing suggestions. **Conclusion:** Multiple clinical interventions were made by the GEMITH pharmacist, which prevented possible and significant medication-related harm. This demonstrated the quality impact of the specialist pharmacist in improving medication safety for geriatric patients.

## Introduction

It is estimated that one-quarter of Australians will be aged 65 years or older by 2045, which is double that of the present population in 2023.^1,2^ This will mean that there is an increasing number of older adults with higher levels of medical complexity, resulting in higher medication use.^3,4^ Increased life expectancy, coupled with multimorbidity, frequently leads to polypharmacy and an increased risk of medication-related harm.^3,4^ As older adults are often at a higher risk of medication harm, they require comprehensive education regarding medications; this is especially needed during transitions of care where there is a greater likelihood of medication errors and harm.^5-7^ Higher consumer expectations of high-quality health care, along with advances in treatment options, necessitate an increasing demand for specialist geriatric services across Australia.^7,8^ Hospital in the home (HITH) programs are one initiative to improve quality of care, and to reduce bed pressure on the inpatient hospital system which mitigates the rising cost of health care.^9^ To meet the growing need for specialist geriatric services, Geriatric HITH models utilise a multidisciplinary approach that combines the HITH model with a Geriatric Evaluation and Management approach. This approach comprises of comprehensive geriatric assessments, action plans for medications, capacity and risk assessments.

In October 2020, A Geriatric Evaluation and Management in the Home (GEMITH) service model was implemented at the Princess Alexandra Hospital (PAH), a quaternary hospital in Queensland, Australia, to target the vulnerable aging population and to improve transitions of care into the community for older adults. The GEMITH model connects patients with appropriate community services, contributes to medication management and optimisation, and ultimately aims to prevent hospital re-admissions. The GEMITH model combines specialist geriatric care with HITH, which is a service proven to reduce mortality, re-admission rates and costs, whilst increasing patient and care satisfaction.^9,10^ The GEMITH service team is multidisciplinary, consisting of a senior geriatrician, a registrar, an occupational therapist, a physiotherapist, a pharmacist, a social worker, an allied health assistant, and clinical nurses. The GEMITH team provides comprehensive geriatric assessment and helps to facilitate patient transitions from the hospital to the community by ensuring that the home environment is safe, and that the patient has ongoing care services in place. This is operationalised by establishing links in with community services if deemed necessary (eg., referrals to Queensland Civil and Administrative Tribunal, My Aged Care, and Non-government organisations) and by utilising members of the GEMITH team to perform services at home (eg., physiotherapy sessions or occupational therapy home based assessments). The GEMITH initiative is unique as it incorporates a specialist pharmacist into the team, with expertise in geriatric care, to optimize medication outcomes.

The benefits of incorporating pharmacists into health care teams to reduce medication harm have been clearly demonstrated within a range of clinical areas and healthcare settings.^11-13^ These benefits have included reducing medication errors during transition of care, detecting and addressing medication related problems, and providing patients and their families or carers with verbal and written medication information. The provision of medication information has led to improvement in patient engagement with the management of their medication therapy and outcomes of their treatment, in collaboration with the prescriber.^14^ In the GEMITH model, the specialist pharmacist plays a key role in ensuring optimal therapeutic choices, with clear documentation of medication changes at each stage of the patients’ hospital journey.^15^ Furthermore, they play an essential part in coordinating and communicating medication changes to primary healthcare providers to ensure that medication plans are reviewed on discharge from the service.^16,17^ While there has been some evidence that HITH pharmacist services may reduce unplanned readmissions,^11^ there is limited evidence on the potential impact of GEMITH pharmacists who are uniquely placed to optimise geriatric services.

## Aim

This study evaluated the type, and potential clinical significance, of specialist geriatric pharmacist interventions, to determine the medication safety and quality impact of the GEMITH service.

## Ethics Approval

Ethics exemption for this study was obtained from the Medicines Use Evaluation Committee at the Princess Alexandra Hospital (CM0504202301).

## Methods

This was a retrospective observational study of clinical interventions made by the GEMITH pharmacist for patients admitted to the service between October 2020 to April 2021.

### Setting and Participants

This study was conducted at the PAH, which is a 1052-bed quaternary hospital in Brisbane, Queensland, Australia. Any patient admitted to GEMITH service during the study period, who had one or more interventions documented by the specialist pharmacist, were included in the study. The time frame from October 2020 to April 2021 was purposely selected to coincide with the implementation of the GEMITH initiative at the PAH, enabling insights into the pharmacists’ contribution at the point of inception of this novel service.

### Data Collection

Pharmacist records of interventions made were manually extracted from the integrated electronic Medical Record (ieMR) for analysis. Pharmacist activities such as medication counselling, admission histories, home visits, daily chart reviews and webster pack (ie, multi-dose medication blister pack) updates or initiations were also collected from the ieMR. The number of medications at admission and discharge was extracted from the Enterprise-wide Liaison Medication system (eLMS). Other key data points collected included demographic and clinical characteristics such as sex, age, and Charlson Comorbidity Index (CCI). Presentation characteristics, such as length of stay (LoS), were obtained from the hospital’s Health Information Management Service.

### Data Analysis

Patient demographic information, clinical characteristics, LoS, and pharmacist activities were descriptively analysed. All interventions recorded by the pharmacist over the study period were rated for their clinical significance using a validated tool by the Society of Hospital Pharmacists of Australia (SHPA) risk classification system (see Supplementary file 1). This tool uses a consequence and probability matrix to classify the risk of the intervention and potential medication-related harm. The risk of medication-related harm of each intervention was classified into five categories to determine the impact of each intervention as ‘insignificant, minor, moderate, major or catastrophic’. Likelihood was assessed as either ‘almost certain, likely, possible, unlikely or rare’. These ratings for consequence and likelihood were used to determine an overall risk rating of clinical significance, which was either classified as ‘low, moderate, high or extreme’. To guarantee quality and consistency, the risk rating assessment for each intervention was conducted individually by three pharmacists: a senior clinical pharmacist and two research pharmacists, who were independent of the GEMITH team. Following individual assessment of the interventions, final ratings were determined by discussion through group consensus meetings. An example of each intervention type according to the risk categories was tabulated to provide context for the types of interventions that were classified in each risk category. A narrative analysis was conducted by grouping the interventions into five key themes as derived through group consensus.

## Results

### Demographic and Clinical Characteristics

There was a total of 119 admissions to the GEMITH service during the study period. Patients had an average age of 83 years and were predominantly females (63%) (Table 1). The median LoS for patients admitted to the GEMITH service during the study period was eight days. GEMITH patients had a median of 10 medications on admission and 12 medications on discharge, demonstrating the relatively high medication burden within this patient population. From these 119 admissions, there were 132 clinical interventions made by the GEMITH pharmacist.

**Table 1.** Demographic Characteristics and Clinical Characteristics of Patient Cohort.

	Median (n = 119)	IQR
Age	83	78-88
Length of stay	8	5-11
Charlson comorbidity index	1	0-2
Number of medicines at admission	10	6-15
Number of medicines at discharge	12	6-15
	Proportion (%)	
Gender (Males)	37	

Key: IQR = Interquartile range.

### GEMITH Pharmacist Service and Activities

The GEMITH pharmacist service provided various clinical activities during the study period. This included 128 medication counselling activities, which were undertaken via telephone or in-person delivery. There were 94 comprehensive medication reviews and 349 daily chart reviews of medications completed by the GEMITH pharmacist during the study period. The GEMITH pharmacist provided a total of 94 home visits and organised 37 webster pack initiations or updates for patients admitted to the GEMITH service.

### Risk Classification of Pharmacist Interventions

The majority (49%) of interventions made by the GEMITH pharmacist were considered as low risk (Table 2). These low-risk interventions comprised of minor charting inconsistencies (e.g., unintentional discrepancy where home medicine oral terbinafine was not charted), requirements for vitamin supplementation, and recommendations for bloods or monitoring. Approximately 27% of interventions were classified as moderate risk and 21% were classified as high risk. Some examples of these interventions included dosing adjustments (e.g., titration of levetiracetam). Only 2% of interventions were classified into the extreme category, which both involved high-risk anticoagulant medications. Some specific examples of pharmacist interventions according to risk classification were described (Table 2).

**Table 2.** Risk Categorization and Description of GEMITH Pharmacist Interventions and Activities.

Intervention Risk Category	Number (%) of Interventions within Each Category
	Example Description of Intervention within Each Category
Extreme	2%
	Investigation into management of enoxaparin – minimal documentation, noted high risk of bleed if aXa level is 1-2 but risk of clotting if aXa is < 1-2. Recommend VASC or HAEM are consulted for ongoing plan (duration plan currently: Lifelong)
High	21%
	Patient has serious issues managing their insulin administration (eg, injects into her finger) and BSLs poorly managed as a result. Treating team to consider ceasing insulin and increasing metformin dose and adding linagliptin for better diabetes management.
Moderate	27%
	Patient transferred home on metformin 2g daily. Recommend this is reduced to 1g daily due to impaired renal function (1g daily appropriate for CrCl of 53 mL/min)
Low	49%
	Vitamin B12 level was 179 on admission, not supplemented on ward, GEMITH treating team please review and consider supplementing.
Total interventions	100%

Pharmacist activities	Number of activities provided by the GEMITH pharmacist from October 2020 to April 2021
Medication counselling (phone or in-person)	128
Medication admission history	94
Home visit	29
Daily chart review	349
Webster pack organised or updated	37
Total activities	637

### Narrative Description of Intervention Types

**Figure 1.**
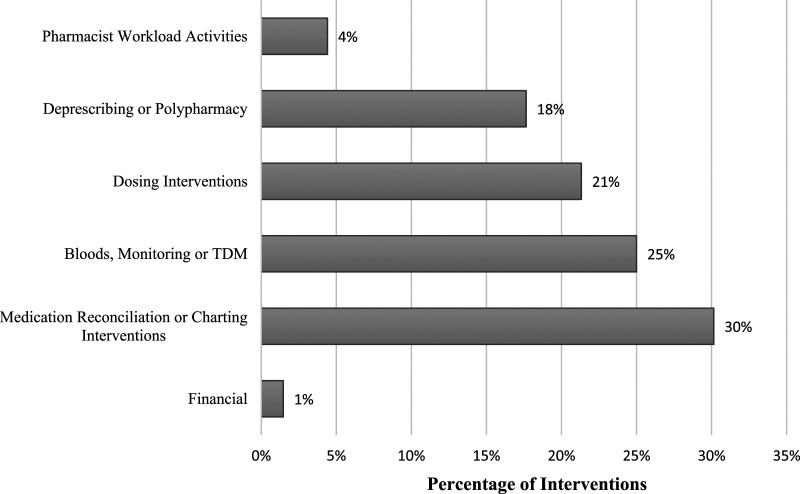
Geriatric Evaluation and Management in the Home Pharmacist Interventions by category type.

There were six key categories of interventions identified (Figure 1). The most common type of intervention (30%) made by the GEMITH pharmacist was related to medication reconciliation or charting interventions. This was mostly a result of transition of care issues, where medications changes were not accurately being reflected on the medication administration record following discharge. The next most common intervention type (25%) was those related to bloods, monitoring or therapeutic drug monitoring (TDM). These interventions were mostly related to recommendations for modification of therapy based off blood tests and commencement of Vitamin D or B12 supplementation based on blood test results. Other intervention types included dosing interventions, deprescribing or polypharmacy, pharmacist workload activities, and financial interventions. Examples of dosing interventions included incorrect dose based on age, weight, or renal function; suboptimal dosing for drug-drug or drug-disease interactions; and dose optimisation for specific indications. Deprescribing or polypharmacy interventions were a key component of medication management approaches in older adults.

## Discussion

### Statement of Key Findings

This study investigated the quality impact of the GEMITH service by evaluating the type and potential clinical significance of specialist geriatric pharmacist interventions. Multiple clinical interventions and activities were performed by the GEMITH pharmacist. Approximately 23% of all interventions were rated as either high or extreme risk, and 26% were rated as moderate risk, demonstrating prevention of potentially significant medication-related harm. Moderate to extreme risk interventions were mainly related to the management and monitoring of high-risk medications such as insulin and anticoagulants. The low-risk interventions were mostly related to medication reconciliation on admission and discharge. Low-risk interventions were an important component of the GEMITH service in optimising medication management, and organising accurate transfer of medication records and plans during transitions of care. Overall, the GEMITH pharmacist improved medication safety through prevention of potential medication related harm.

### Benefits of Incorporating Pharmacists into HITH Services

The incorporation of a pharmacist into HITH services is an opportune point in the patient’s journey for review of medications, and the benefits to medication safety of HITH pharmacists are well established. A study investigating pharmacist involvement in a mental health HITH service in Western Australia reported significant improvements in key medication safety performance indicators.^14^ They reported higher rates of completed medication reconciliation, accurate discharge medication lists, and increased medication chart reviews.^14^ These findings were consistent with this study, where the majority of GEMITH pharmacist interventions focused on medication reconciliation that led to correction of unintended charting discrepancies. Pharmacist involvement in medication reconciliation can improve the accuracy of medication lists, which often have inconsistencies that occur during transitions of care. This means that comprehensive medication reviews can also improve clinical outcomes and patient safety. This is supported by a systematic review and meta-analysis which found that pharmacist-led medication reconciliation improves patient safety through prevention of potential medication harm.^18^ Previous studies report over 50% prevalence of error in medication reconciliation post discharge from hospital, an outcome that resonates with a GEMITH model of care that involves a pharmacist reconciling a discharge medication list.^19^ Further to this, patients admitted to the GEMITH service had been transferred through other hospital wards with pharmacist reviews during their patient journey. This highlights the success of incorporating a pharmacist into the GEMITH service to identify issues that might have been missed at earlier transfers of care. As the GEMITH service occurs in the patient’s home, as opposed to traditional inpatient care in hospital, there are potential cost reductions to the hospital and health care service.^20^

### Importance of Multidisciplinary Geriatric Care

Pharmacists have unique and specialised medication knowledge, which they can independently use to enhance patient care and contribute to the multidisciplinary team.^21^ A 2019 Canadian study that explored inclusion of a pharmacist in a multidisciplinary service found positive impacts on medication management in the family medicine setting, observed through detection and resolution of medication-related problems, and reduction in medication regimen complexity and non-adherence. A key finding from this Canadian study was that the acceptance of pharmacist recommendations was higher when they were closely integrated with the team. This demonstrates the importance of embedding a specialist geriatric pharmacist into the GEMITH team and service. While this study did not investigate relationships between the team, an inferred benefit can be realized through the interventions made by the GEMITH pharmacist and their active involvement in multidisciplinary team meetings and contribution to patient treatment plans. In this study, the GEMITH pharmacist identified and resolved 28 high risk interventions and 2 extreme risk interventions, reducing the potential for significant medication harm to patients admitted to the GEMITH service. Providing multidisciplinary geriatric care also means that pharmacists can support other clinicians and assist in clinical decision making. Pharmacists undertaking medication histories and medication reconciliation can free up time for other clinicians who may have been previously completing these activities prior to pharmacist involvement in the team. This demonstrates potential reductions in workload pressures for the entire GEMITH service. Another study within a residential aged care setting supported this benefit as they reported that two hours of pharmacist time freed up three hours of nursing time and one hour of physician time.^22^

### Specialist Geriatric Pharmacist to Optimise Outcomes for Older Adults

The incorporation of a pharmacist with specialist knowledge in geriatric care within the HITH service is integral to optimising outcomes for older adults. A study by Leguelinel-Blache et al^23^ highlighted that unintended medication discrepancies were more common in older patients with a greater degree of polypharmacy, necessitating proactive medication reconciliation. This means that pharmacists are uniquely placed to provide medication management and optimisation within specialist geriatric services, such as within the GEMITH model of care. Leguelinel-Blache et al^23^ reported that the older adult population (mean age of 83 years) had over 10 medications on average, highlighting the real need for medication reconciliation and monitoring as an essential component of the GEMITH service. Comprehensive geriatric assessments (CGA) is an evidence based, interdisciplinary assessment of the older patient, of which medication management is a recognised core domain. Reduction in polypharmacy, dose optimisation, and therapeutic monitoring is a key focus of medication management during CGA. The addition of a specialised pharmacist into the GEMITH service helped enhance medication management within the CGA framework. Other studies, across multiple settings, have further demonstrated the addition of pharmacists in completing medication management activities, reducing polypharmacy, and decreasing use of potentially inappropriate medicines.^6,7,10^ This study also highlighted areas where clinicians can improve patient management in a geriatric population. Common interventions made by the GEMITH pharmacist were related to bloods, monitoring or TDM (26%), as well as dosing interventions (22%), and deprescribing or polypharmacy (18%). Bloods, monitoring or TDM interventions were often related to the monitoring of subtherapeutic vitamin D or B12 levels, which is essential for geriatric care, and was either not completed or actioned. Pharmacists play an important role in recognising the need for dose reductions because of age, weight or renal function, which is common in the older adult population. For example, digoxin was often flagged as a drug for potential dose reduction by the GEMITH pharmacist. Pharmacists also play an active role in promoting deprescribing where appropriate. A significant proportion (19%) of the interventions in this study were related to deprescribing of aspirin or statin medications, as indicated for primary prevention. Removing potentially inappropriate medications can prevent the potential for medication interactions and medication-related harm. Lastly, pharmacists provide a myriad of other activities outside of interventions. Daily chart reviews, medication counselling, and organization of webster packs (ie, multi-dose medication blister pack) for discharge are essential components of medication management that are often overlooked or not completed.

## Strengths and Weaknesses

Some of the main limitations of this study included the potentially smaller sample size and shorter study period. As this study was undertaken in a large quaternary hospital in a metropolitan area, there is a potential limitation in the generalisability of the findings to other settings. However, learnings and opportunities of incorporating a pharmacist into geriatric services can be translated to other contexts. One limitation of this study included the absence of data collection on pharmacological groups or classes of drugs, which would have given further insights into GEMITH service activity. While CCI was collected and reported, this may not have been the most useful measure for this population, given that it is based on specific diagnoses which may not have been present in this cohort. While this research found that there were multiple pharmacist interventions made, no data was collected on endpoints, such as readmission rates or clinical outcomes. Future research should incorporate measurement of outcomes beyond pharmacist interventions, such as mortality or preventable readmissions. However, the use of pharmacist interventions does show the potential prevention of medication related harm. As this study was retrospective, there is a potential for incomplete data which could have impacted the results presented, and it is possible that some interventions made by pharmacists occurred but were not documented. A major strength of this study was that it observed real-world pharmacist involvement in a novel service for geriatric care. The interventions were categorised according to a well-established and validated tool and were reviewed independently by three pharmacists. This contributed to great quality and consistency of the risk rating process, adding to the robustness of the method used.

## Conclusion

This study found that the GEMITH pharmacist improved medication safety through the prevention of potentially significant medication related harm. This study highlighted the benefits of incorporating pharmacists into HITH services, and the importance of providing multidisciplinary geriatric care. The incorporation of a pharmacist with skills and knowledge in specialist geriatric care can help optimise medication outcomes for older adult patients. Older adults often have greater polypharmacy and an increased need for medication management and optimization, which means that pharmacists can be uniquely placed to improve care in these populations. The addition of a specialist pharmacist into the GEMITH service can further enhance the provision of comprehensive geriatric assessments provided during hospital admissions. The findings from this study could serve as a roadmap for future implementation and evaluation of other GEMITH services across hospitals and healthcare services in Australia.

## Supplemental Material

Supplemental Material - Assessment of a Geriatric Evaluation and Management in the Home (GEMITH) Service at a Quaternary Hospital: A Retrospective Observational Study Supplemental Material for Assessment of a Geriatric Evaluation and Management in the Home (GEMITH) Service at a Quaternary Hospital: A Retrospective Observational Study by Keshia R. De Guzman, Duncan Long, Bpharm, Alexander Theodos, Alexandra Karlovic, and Nazanin Falconer in Journal of Pharmacy Practice.
