# Supplementary material for: Assessment of a Geriatric Evaluation and Management in the Home (GEMITH) Service at a Quaternary Hospital: A Retrospective Observational Study
Source: J Pharm Pract. 2024 Jun 13;38(1):28–34. doi: 10.1177/08971900241262376 (PMC11639412; doi:10.1177/08971900241262376)
Supplement: Supplemental Material - Assessment of a Geriatric Evaluation and Management in the Home (GEMITH) Service at a Quaternary Hospital: A Retrospective Observational Study [file sj-pdf-1-jpp-10.1177_08971900241262376.pdf]

Supplementary files

Supplementary file 1: Validated risk rating tool – consequence/probability matrix

**Consequence of impact**		
**Level**	**Descriptor**	**Description:** assume intervention not made, probable scenario (not worst case)
1	Insignificant	No harm or injuries, low financial loss
2	Minor	Minor injuries, minor treatment required, no increased length of stay or re-admission, minor financial loss
3	Moderate	Major temporary injury, increased length of stay or re-admission, cancellation or delay in planned treatment/procedure. Potential for financial loss
4	Major	Major permanent injury, increased length of stay or re-admission, morbidity at discharge, potential for significant financial loss
5	Catastrophic	Death, large financial loss and/or threat to goodwill/good name
**Likelihood of occurrence**		
**Level**	**Descriptor**	**Description:** likelihood of impact occurring without intervention and scenario occurring in the future
A	Almost certain	Is expected to occur in most circumstances
B	Likely	Will probably occur in most circumstances
C	Possible	Might occur at some time
D	Unlikely	Could occur at some time
E	Rare	May only occur in exceptional circumstances

**Risk (consequence x likelihood)**					
**Likelihood**	**Insignificant**	**Minor**	**Moderate**	**Major**	**catastrophic**
A (almost certain)	H	H	E	E	E
B (likely)	M	H	H	E	E
C (possible)	L	M	H	E	E
D (unlikely)	L	L	M	H	E
E (rare)	L	L	M	H	H
E=extreme-risk, H= high-risk, M=moderate-risk, L=low-risk					

Supplementary file 2: Description of Intervention Categories

Deprescribing or Polypharmacy

This applies to any intervention where the clinical pharmacist has recommended deprescribing or ceasing a medication due to polypharmacy, cognitive decline, lack of evidence or to simplify a medication regime and promote compliance.

Dosing interventions

This category is applied to any interventions pertaining to the dose or frequency of a medication. The clinical pharmacist has made specific recommendations for dosing based on age, bodyweight, renal function or drug-drug interactions.

Bloods, Monitoring or TDM

In this category the clinical pharmacist has recommended bloods to be done as part of appropriate monitoring for new/continuing therapy (ie. Vitamin D/Calcium for Denosumab therapy). This category is also applied if the clinical pharmacist has recommended initiation of therapy based on blood results (ie. B12 loading for vitamin B12 deficiency).

Medication Reconciliation or charting interventions

These are interventions related to discrepancies between the discharge reconciliation plan on transfer to GEMITH and the medications charted on the medication chart, as often medications will be changed on discharge, but it is not common practice to update the medication chart with these changes (as the medication chart usually becomes obsolete on discharge). Clinical pharmacist interventions ensure the medication chart is accurate, so changes to medications are clinically appropriate.

Financial

This may relate to the cost of a medication to the hospital – particularly if it is a medication not on the list of approved medications (LAM). This also may relate to the cost of a medication to the patient – if the cost of a medication going to impact ongoing therapy.
